# Graphene-based mid-infrared room-temperature pyroelectric bolometers with ultrahigh temperature coefficient of resistance

**DOI:** 10.1038/ncomms14311

**Published:** 2017-01-31

**Authors:** U. Sassi, R. Parret, S. Nanot, M. Bruna, S. Borini, D. De Fazio, Z. Zhao, E. Lidorikis, F.H.L. Koppens, A. C. Ferrari, A. Colli

**Affiliations:** 1 Cambridge Graphene Centre, University of Cambridge, Cambridge CB3 0FA, UK; 2 ICFO Institut de Ciencies Fotoniques, The Barcelona Institute of Science and Technology, 08860 Castelldefels, Barcelona, Spain; 3 Nokia Technologies, Broers Building, Cambridge CB3 0FA, UK; 4 Department of Materials Science and Engineering, University of Ioannina, Ioannina 45110, Greece; 5 ICREA Institució Catalana de Recerça i Estudis Avancats, Barcelona 08010, Spain; 6 Emberion Ltd, Sheraton House, Castle Park, Cambridge CB3 0AX, UK

## Abstract

There is a growing number of applications demanding highly sensitive photodetectors in the mid-infrared. Thermal photodetectors, such as bolometers, have emerged as the technology of choice, because they do not need cooling. The performance of a bolometer is linked to its temperature coefficient of resistance (TCR, ∼2–4% K^−1^ for state-of-the-art materials). Graphene is ideally suited for optoelectronic applications, with a variety of reported photodetectors ranging from visible to THz frequencies. For the mid-infrared, graphene-based detectors with TCRs ∼4–11% K^−1^ have been demonstrated. Here we present an uncooled, mid-infrared photodetector, where the pyroelectric response of a LiNbO_3_ crystal is transduced with high gain (up to 200) into resistivity modulation for graphene. This is achieved by fabricating a floating metallic structure that concentrates the pyroelectric charge on the top-gate capacitor of the graphene channel, leading to TCRs up to 900% K^−1^, and the ability to resolve temperature variations down to 15 μK.

## Abstract (web summary)

There is emerging interest in photodetectors in the mid-infrared driven by increasing need to monitor the environment for security and healthcare purposes. Sassi *et al*. show a thermal photodetector, based on the coupling between graphene and a pyroelectric crystal, which shows high temperature sensitivity.

Detecting thermal infrared (IR) radiation of room temperature (RT) objects (with spectral peak emittance ∼10 μm (refs 1, 2)) is increasingly important for applications in astronomy^3^, healthcare^4^,^5^, smart energy systems^6^, security^7^, pollution monitoring^8^, fire sensing^9^, automotive^10^ and motion tracking^11^. In this spectral region, thermal photodetectors (PDs) that can operate at RT with no need for cooling are highly desirable^2^,^12^.

Pyroelectric detectors are low-cost, uncooled thermal PDs for the mid-infrared (MIR)^1^,^2^,^12^. They are capacitor-like structures where a pyroelectric crystal is sandwiched between two metal electrodes^2^. Pyroelectric crystals are materials with a *T*-dependent spontaneous polarization, *P* (C m^−2^), i.e., surface density of bound charge^12^. Around RT, a linear relation links the *T* variation, Δ*T*, with the changes of *P*^1^,^2^,^12^:


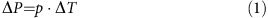


where *p* (μC m^−2^ K^−1^) is the pyroelectric coefficient (for the crystallographic direction perpendicular to the electrodes). The two metal electrodes are connected through an external load resistor *R*_L_. At thermal equilibrium (d*T*/d*t*=0), no current flows in the external circuit, because *P* is constant and the charges on the electrodes compensate the bound charges at the pyroelectric surface^12^. However, when the detector is illuminated, the absorbed radiation heats the crystal and *P* changes according to equation (1)^12^. The variation of the bound charge surface density will induce a current *I*_p_ in the external circuit^12^:


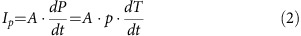


where *A* (m^2^) is the electrode area^1^,^13^. *I*_p_ flows only as long as *T* changes (i.e., when the impinging optical power changes).

Bolometers are another class of uncooled thermal PDs, where *T* variations due to incoming photons produce a change in the resistance (*R*) of a sensing element. This can be a thin metal layer^14^, a semiconductor^15^ or a superconductor^1^. Common metallic bolometers for RT operation are made of Ti^16^, Ni^17^ or Pt^14^. Polysilicon^1^,^2^, amorphous silicon^18^ or vanadium oxide^15^ are usually exploited for semiconducting bolometers. For fixed bias, *V*_d_, the resistance change of the sensing element translates in a measurable change in current (*I*). The temperature coefficient of resistance (TCR in units of % K^−1^) is a key performance indicator for a bolometer and is defined as^2^:


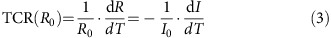


The TCR represents the percentage change in resistance per Kelvin around the operating point *R*_0_ and corresponds in module to the normalized current change per Kelvin around the operating current *I*_0_ (Equation 3). The TCR in metallic bolometers is ∼0.4% K^−1^ (ref. 2), whereas for semiconducting bolometers it is ∼2–4% K^−1^ (refs 1, 2). It follows that the output of a bolometer (measured current) is proportional to *T*, in contrast to the output of a pyroelectric detector (measured current) that depends on the derivative of *T*, see equation (2)^13^. However, although the TCR of a bolometer does not depend on the device area, in pyroelectric detectors the output current is a function of the electrode size, as for equation (2)^13^. Larger electrodes allow the collection of more charge, increasing the pyroelectric current, therefore leading to a larger signal.

These differences have an impact on the suitability of both technologies for different applications. As pyroelectric detectors are alternating current devices that rely on a variable impinging radiation, they require a chopper at 25–60 Hz^12^ to detect stationary objects and are thus preferentially used to detect moving targets (e.g., for automatic lighting systems^6^, electrical outlet turn-off^11^, unusual behaviour detection^19^, home invasion prevention^12^ and so on), where they are not only able to detect the presence of warm bodies^2^ but also to extrapolate parameters such as distance, direction or speed of movement^11^. Such information can be obtained by processing the analogue signals of only a couple of large (∼1 cm^2^) detectors^11^. On the other hand, bolometers can be scaled to smaller sizes without any loss in TCR to make arrays of pixels for stationary imaging. Resistive micro-bolometers used in high-resolution thermal cameras range from 17 × 17 to 28 × 28 μm^2^ in size^2^.

Graphene is ideally suited for photonic and optoelectronic applications^20^,^21^,^22^, with a variety of PDs in the visible^23^,^24^,^25^,^26^,^27^, near-infrared (NIR)^21^,^22^ and THz reported to date^28^, as well as MIR thermal detectors^29^,^30^,^31^. 32,33,34 previously reported graphene-based bolometers at low *T* (<10 K). However, these are not viable for practical applications in the mass market, where RT operation is needed. At RT, single-layer graphene (SLG) is not competitive as the sensing element of a bolometer, as it shows a maximum TCR ∼0.147% K^−1^ (ref. 35), lower than both the metallic and semiconducting bolometers discussed above. 36 used reduced graphene oxide films with TCR ∼2.4–4% K^−1^ at RT, whereas (37) exploited vertically aligned graphene nanosheets to produce infrared bolometers with TCR ∼11% K^−1^ at RT, the largest, to date, for carbon nanomaterials^37^. In these films, however, conduction is modulated by thermally assisted hopping between different sheets or localized defect sites^36^ and not by any intrinsic property of SLG.

SLG, however, can play a key role when integrated on polarizable materials (pyro, piezo or ferro-electric)^38^,^39^,^40^,^41^. For example, SLG can be used as a transducer for the pyroelectric polarization due to its field-effect response^38^. If a SLG field-effect transistor (GFET) is fabricated on a pyroelectric substrate, the channel resistance is modulated by the substrate polarization and can thus represent a direct *T* readout. This is the electrical equivalent of a bolometric response with area-independent TCR (the charge density of the pyroelectric ‘gate' is constant and does not depend on the size and shape of the SLG channel). 38 reported a TCR ∼6% K^−1^ for GFETs on lead zirconate titanate, a material with one of the largest pyroelectric coefficient known to date (up to 780 μC m^−2^ K)^42^. This indicates that the pyroelectric charge density generated by lead zirconate titanate underneath the SLG channel does not yet allow to outperform state-of-the-art bolometers^38^.

Here we demonstrate a RT PD for MIR by integrating a dual-gate SLG amplifier with a pyroelectric material. As this is a two-terminal device whose resistance changes proportionally to *T*, mimicking the intrinsic material property of a bolometric resistor^2^, we can measure an ‘effective' TCR as a metric for its net electrical output. Internally, the PD comprises a floating metallic structure that concentrates the charge generated by the pyroelectric substrate over an integrated GFET. As charge cannot escape from the floating structure (i.e., there is no load resistor), the PD can be operated in direct current (dc) and there is no need for chopping. We call this structure ‘graphene-based pyroelectric bolometer', as it combines a pyroelectric sensor with a SLG transducer to deliver a dc bolometric response. We emphasize that the proximity of the SLG amplifier is essential to minimize the parasitic capacitances that would remove the pyroelectric charge, should the FET amplifier be implemented as an external separate component. The total pyroelectric charge generated on a variation in *T* increases with area, delivering effective TCRs up to 900% K^−1^ for a footprint of 300 × 300 μm^2^, i.e., two orders of magnitude larger than state-of-the-art IR PDs having any similar or larger area^1^,^2^,^36^,^37^. The TCR scaling is sub-linear for smaller footprints. We discuss the origin of this behaviour and conclude that our device performance is competitive even in the limit of small pixels (∼10 × 10 μm).

## Results

### Device architecture

**Figure 1:**
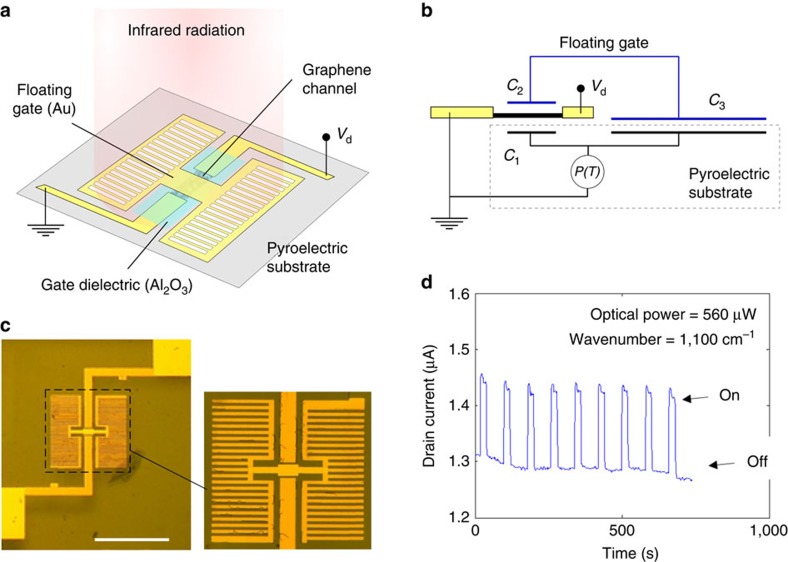
Graphene pyroelectric bolometer. (**a**) Scheme of an individual device, where the conductance of a SLG channel is modulated by the pyroelectric substrate and by a floating gate. This is driven by two metallic pads in contact with the substrate, with a total area much larger than the overlap with the SLG channel. Such pads can be either uniform or patterned. (**b**) Circuit diagram for the device in **a**. (**c**) Optical image of a device with lateral pads patterned as electrically connected finger-like structures. Scalebar, 300 μm. (**d**) Response at 1,100 cm^−1^ (∼9 μm) over several ON/OFF cycles induced by a manual shutter. The laser spot size is 300 μm. The drain current is measured for a 10 mV drain voltage (*V*_d_).

Figures 1a,b show the layout of a single device and the corresponding electrical model. A SLG channel with source and drain contacts is fabricated on the pyroelectric substrate (500 μm-thick *z*-cut lithium niobate (LN)) as described in Methods. A 10 nm-thick Al_2_O_3_ dielectric layer isolates the SLG from an H-shaped floating Au structure designed to overlap the oxide-coated SLG in the centre, whereas lateral pads are placed in direct contact with the substrate. Both uniform and patterned Au pads have been studied, the latter in the form of finger-like structures (to enhance light absorption at selected wavelengths, see Supplementary Note 2). The design is such that the SLG channel conductivity can be modulated by a dual-gate capacitive structure. From the bottom, there is the pyroelectric polarization (and associated electric field) generated directly by the substrate (*C*_1_ in Fig. 1b), which we refer to as the ‘direct effect' on SLG conductivity (previously exploited in ref. 38). From the top, there is a gate *C*_2_ connected in series with capacitor *C*_3_ as a floating circuit branch, with *C*_3_>*C*_2_. The perimeter of the pads defining *C*_3_ sets the overall pixel size, from which only the source and drain contacts stem out to interface with the measurement electronics.

In first approximation, the generated pyroelectric charge Δ*Q* is uniformly distributed on the substrate on a *T* variation^1^,^2^. Therefore, the direct effect from *C*_1_ does not depend on the channel area *A*_C1_, as the bottom-gate field depends on the pyroelectric polarization, which is constant over any area. For the floating gate in Fig. 1b, Δ*Q* accumulating on *C*_3_ depends on area as (from equation (1)) Δ*Q*=*p*Δ*TA*_C3_. Being the structure electrically floating and free from external parasitic capacitances, Δ*Q* is entirely provided by *C*_2_, because of the conservation of charge. A charged *C*_2_ generates for the SLG channel an effective top-gate voltage (in module):


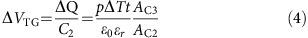


where *C*_2_=*ɛ*_0_ *ɛ*_r_ *A*_C2_ *t*^−1^, *ɛ*_0_ and *ɛ*_*r*_ are the vacuum and relative permittivity, and *t* is the oxide thickness. Hence, for fixed *t* and Δ*T*, the geometrical ratio *A*_C3_/*A*_C2_ controls the gain of the integrated SLG amplifier and therefore the TCR (Δ*I I*^−1^=*g*_m_ Δ*V*_TG_ *I*^−1^, where *I* is the current and *g*_m_ is the transconductance of the GFET).

Figure 1c shows an optical micrograph of a device with patterned pads. We illuminate this device with MIR radiation at 1,100 cm^−1^ (∼9 μm) using a laser spot matching the pixel size (300 × 300 μm^2^). The resulting modulation of the channel drain current is shown in Fig. 1d over nine ON/OFF laser cycles. A responsivity ∼0.27 mA W^−1^ is obtained, for a drain current in the dark (*I*_OFF_) ∼1.3 μA (*V*_d_=10 mV). The responsivity can be increased by applying a larger *V*_d_, which, in turn, increases *I*_OFF_. The normalized current responsivity (% W^−1^) is defined as:^2^


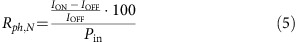


where *I*_ON_ is the current under illumination and *P*_in_ is the optical power of the incoming radiation. *R*_pn,N_ is a better parameter to compare photoconductive detectors. *R*_pn,N_ in Fig. 1d is ∼2 × 10^4^ % W^−1^, over two orders of magnitude higher than ref. 38, where only the direct effect was exploited (∼1.2 × 10^2^ % W^−1^)^38^.

### Photomapping and wavelength dependence

**Figure 2:**
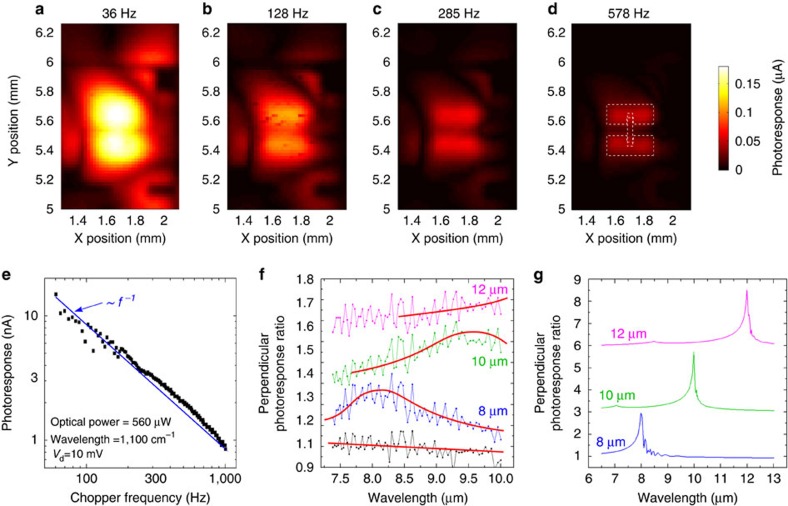
Optical response. (**a**–**d**) Photocurrent profiles of a representative device for different *f* and a beam of 1.8 mW at 1,100 cm^−1^. The peak intensity decreases at higher *f*, but the map is more resolved. The dashed white lines in **d** indicate the location of all device features, rotated 90° with respect to Fig. 1c. (**e**) Dependence of the locked-in photoresponse with *f* for a fully illuminated device (laser diameter=300 μm), showing *f*^−1^ scaling above 60 Hz. (**f**) Wavelength dependence of the photoresponse for a device patterned as in Fig. 1c. The pitch of the fingers varies between 8 and 12 μm. Peaks are observed in the photoresponse ratio between parallel and perpendicular polarized light and their position changes with pitch (the red lines are guides to the eye). The flat photoresponse ratio for a device with uniform Au pads (black data points) is also shown as reference. (**g**) Simulated total absorption for the devices measured in **f**. Predicted peaks in the parallel/perpendicular absorption ratio match those measured for the photoresponse.

By reducing the laser spot to ∼10 μm and using a lock-in, we produce photocurrent maps of a single pixel to assess where the maximum signal is generated (Fig. 2). At the slowest chopper frequency (*f*=36 Hz; Fig. 2a) the photocurrent map shows two broad peaks (∼700 μm), which largely overlap and extend beyond the pixel area. When *f* is increased, the two peaks become progressively resolved until, above 500 Hz (Fig. 2d), they match the location of the lateral pads defining the pixel. These are not necessarily the areas where the strongest absorption occurs, but those where *T* changes are detected providing the highest photocurrent *I*_ph_ (where *I*_ph_=*I*_ON_−*I*_OFF_). Figures 2a–d also show that the signal decreases at higher *f*. To better quantify this trend, we plot *I*_ph_ on full illumination (300 × 300 μm^2^ spot size; Fig. 2e) as a function of *f*. *I*_ph_ scales linearly with *f*^−1^ and is measurable up to 1 kHz. This PD can be described by a thermal model (see Supplementary Note 1). When an increase in illumination time per cycle (i.e., a reduction in *f*) results into a proportional T increase, the system is far from a dynamic equilibrium with the thermal sink (the chip carrier) within a single cycle. This is the behaviour we observe for *f*>60 Hz. As for slow chopping speeds (Fig. 2a,b) there is more time for heat to laterally spread away from the illuminated spot, the *T* within the pixel becomes more homogeneous, resulting in blurred photomaps.

Figure 2f plots the wavelength dependence of the photocurrent for devices with lateral pads patterned with a finger-like design (as in Fig. 1c) with different pitches. The fill ratio (i.e., Au finger area per total available pad area) is kept constant at 0.5, meaning that, e.g., fingers with an 8 μm pitch are 4 μm wide and separated by a 4 μm gap. Although for a uniform Au pad the photoresponse to parallel and perpendicular light is the same for all wavelengths (black data in Fig. 2f), for patterned pads a peak arises in the parallel/perpendicular photoresponse ratio at the wavelength that matches the fingers pitch.

We then simulate the total parallel/perpendicular absorption for finger-like Au structures on thick LN (Fig. 2g; see Supplementary Note 2 for details). This shows that a peak is expected at the wavelength corresponding to the fingers pitch. This is consistent with Fig. 2f, as more absorption results into a larger *T* increase and therefore a larger signal. In the calculations we assume that all light entering the bulk LN substrate is eventually absorbed, contributing to the *T* rise. The parallel-polarized light gets more absorbed overall (i.e., in the Au fingers plus LN substrate) than the perpendicular-polarized light, despite the fact that at the resonant frequency the perpendicular-polarized light is absorbed more inside the Au fingers. The reason for this is that resonant absorption in the fingers is also accompanied by resonant reflection, which lowers the overall delivery of light into the Au-LN system as 1−resonant reflection. Things would change if these devices were fabricated on pyroelectric layers having a thickness ∼1 μm (rather than a 500 μm-thick LN crystal). The absorption of the resonant structures would become dominant over the intrinsic absorption of the substrate and reflectance would play a minor role. Figure 2f proves that our device layout is well suited for the implementation of photonic structures to engineer photon absorption and that a spectrally selective MIR response is feasible.

The data in Fig. 2 and the thermal model presented in Supplementary Note 1 suggest that better results could be obtained for devices with an optimized thermal management compared with that offered by a 500 μm-thick pyroelectric substrate. Indeed, the PDs in Figs 1 and 2 spend most of the photons to heat the bulk rather than the surface, limiting the overall *T* increase for a fixed incident power. The fabrication of isolated pixels with lower heat capacity, using thin suspended bridges or membranes (with typical thickness ∼1 μm, as routinely done for microbolometers)^2^, would improve the responsivity, speed and wavelength selectivity.

### Thermo-electrical characterization

We now consider the performance as local thermometer, independent from the conversion of photons to *T* (linked to the emissivity and to the thermal properties of the device, such as thermal capacity and thermal conductance)^2^,^13^. Each pixel is a two-terminal device whose resistance represents a readout of the local *T*. As for bolometers^2^, we consider the TCR as the figure of merit. Being a normalized parameter, the TCR does not depend on *V*_d_. For our devices, it depends on pixel area, hence we will link each TCR to the size of the corresponding pixel. Furthermore, as our variations in resistance are the result of a gain mechanism, we consider how such variations compare with the device noise. We introduce the noise equivalent substrate temperature (NEST), i.e., the pixel T change needed to produce a signal equal to the amplitude of the noise. This is not to be confused with the noise equivalent temperature difference^1^, often used to indicate the smallest detectable *T* change in an IR-emitting body imaged by a PD, also dependent on the photon-to-*T* conversion^1^.

**Figure 3:**
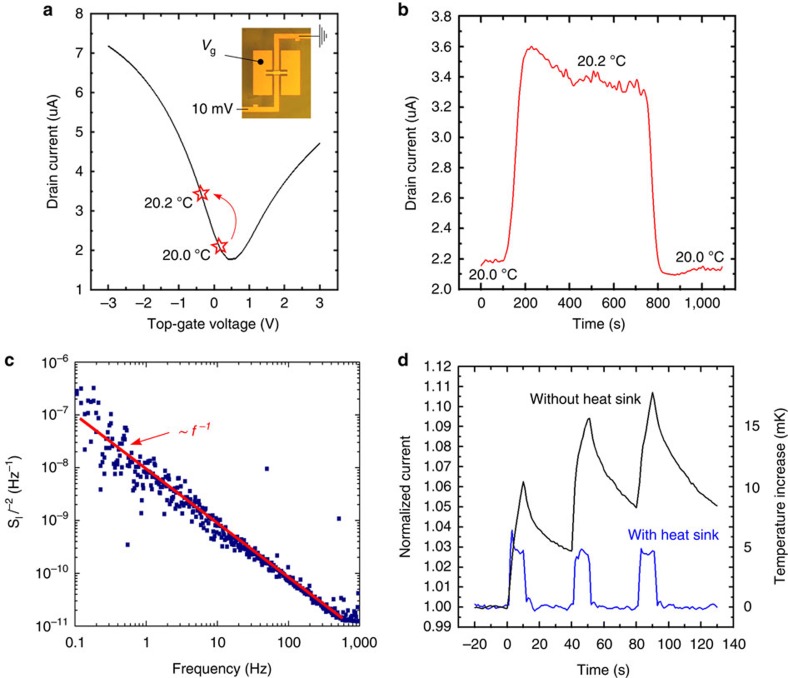
Thermo-electrical characterization. (**a**) Transfer characteristics of a typical device acquired by driving *V*_g._ (**b**) *T* response of the same device under floating gate conditions. Once plotted over the GFET Dirac curve in **a**, the change in drain current shows that a *T* variation of 0.2 °C produces a *V*_g_=−0.44 V (see Supplementary Note 6). (**c**) Normalized noise spectrum density for a representative device at constant *T*, showing the typical *f*^−1^ behaviour for SLG channels. (**d**) Normalized current response of a SLG pyroelectric bolometer to thermal body radiation (human hand at a distance ∼15 cm). The local *T* increase is estimated from the TCR. The *T* transients change in amplitude and speed according to the heat sink efficiency.

In Figs 3a,b we investigate the thermo-electrical characteristics of a representative device (pixel size: 100 × 100 μm^2^, *A*_C3_/*A*_C2_=22) by placing the sample on a chuck with *T* control. In these measurements the sample is in thermal equilibrium in the dark and photon absorption plays no role, thus uniform Au pads are chosen to accommodate (when needed) an electrical probe on the gate pads and apply an external gate voltage (*V*_g_) to the device. Figure 3a shows a typical Dirac curve for a GFET at *T*=20 °C. When an active electrical probe is connected to the gate pad, it acts as a sink neutralizing all the charge generated by the pyroelectric material (*C*_3_) on any *T* change. Because of this, we measure the same transfer characteristics at all *T*, except for the small shift induced by the direct bottom gating (*C*_1_) of the substrate (∼5% K^−1^; see Supplementary Note 3). The vast majority of our devices are slightly *p*-doped (hole density ∼2.5–3 × 10^12^ cm^−2^, in agreement with our Raman data, see Methods), which is the ideal condition to achieve a maximum signal on heating. This is linked to our choice of placing SLG on the positive face of *z*-cut LN, where heating reduces the net dipole moment, equivalent to a negative *V*_g_^12^. We stress that the slightly *p*-type behaviour is the reproducible result of our transfer method, with a ∼80% yield in terms of consistent doping. Because of the passivation offered by the Al_2_O_3_ gate dielectric, the initial SLG doping does not show significant variations over several months (see Supplementary Fig. 8). After removing the gate probe to leave the gate structure floating, we monitor the GFET drain current, while *T* is raised by 0.2 °C, kept constant for 10 min and then decreased to its original value. The resulting plot in Fig. 3b shows that the drain current increases by ∼50% for a 0.2 °C *T* change (TCR∼250% K^−1^), is stable over time and then returns to its original value with negligible hysteresis. The red star markers on the electrically driven Dirac curve in Fig. 3a show how the SLG conductivity evolves when the gate is thermally driven as in Fig. 3b. This stable dc response over several minutes indicates that no appreciable leakage occurs through the pyroelectric crystal and/or the GFET gate within a practical measurement timeframe. The initial bump to 3.6 μA in Fig. 3b is due to the small overshoot of the chuck *T* at the end of the ramp.

To evaluate the NEST we measure the normalized noise power spectrum for a representative device (Fig. 3c). The spectrum is dominated by *f*^−1^ noise up to 1 kHz and closely resembles those previously measured for SLG devices^43^. The channel area (*L* × *W*) normalized noise (*S*_I_ *I*^−2^)(*L* × *W*) is ∼5 × 10^−7^ μm^2^ Hz^−1^ at 10 Hz (considering our 20 × 30 μm^2^ SLG channel), which slightly exceeds the typical range (∼10^−8^–10^−7^ μm^2^ Hz^−1^) reported for SLG devices on SiO_2_ (ref. 43). Considering a pixel size of 100 × 100 μm^2^ and a TCR of ∼214% K^−1^, we get for the device in Fig. 3b a NEST ∼40 μK Hz^−1/2^ at 1 Hz (NEST=(*S*_I_ *I*^−2^)^1/2^ TCR^−1^). For the biggest pixel size (300 × 300 μm^2^, TCR ∼600% K^−1^), the minimum NEST is ∼15 μK Hz^−1/2^. For the MIR PD in Fig. 1, this *T* resolution translates into a noise equivalent power (NEP) ∼5 × 10^−7^ W Hz^−1/2^ at 1 Hz (NEP=(*S*_I_ *I*^−2^)^1/2^ *R*_phN_^−1^), almost one order of magnitude better than that in ref. 44. The associated detectivity at 1 Hz is ∼6 × 10^4^ Jones (D*=*A*^1/2^ NEP^−1^, where *A* is the area of the pixel), which is promising, considering the limitations in terms of thermal conductivity and mass^2^.

To appreciate what these numbers mean in practice, we show in Fig. 3d the photoresponse of a large device (300 × 300 μm^2^, TCR ∼600% K^−1^) illuminated by the IR radiation emitted by a human hand at a distance of ∼15 cm. In one test the sample is placed directly on a large (200 mm diameter) metal chuck (with heat sink, blue data) and in another it is placed in a concave plastic box that keeps it suspended, thus more thermally isolated (without heat sink, black data). Without heat sink, the PD heats up more (hence larger device responsivity), but its response and recovery are much slower. Even with heat sink, the proximity of the hand is easily detected. The saturation signal is ∼3%, corresponding to a *T* increase ∼5 mK. To the best of our knowledge, the only RT SLG detector working at 10 μm and able to allow human hand detection is described in ref. 29. This was fabricated on a suspended and very thin (<1 μm) SiN membrane, measured in vacuum and with a lock-in with 10 s integration time^29^. Here we achieve the same result on a bulk (500 μm-thick) substrate with a resistive measurement in air with 200 ms integration time, indicating that our SLG-based pyroelectric bolometer can provide far better performance (in terms of responsivity and speed) in equivalent conditions.

**Figure 4:**
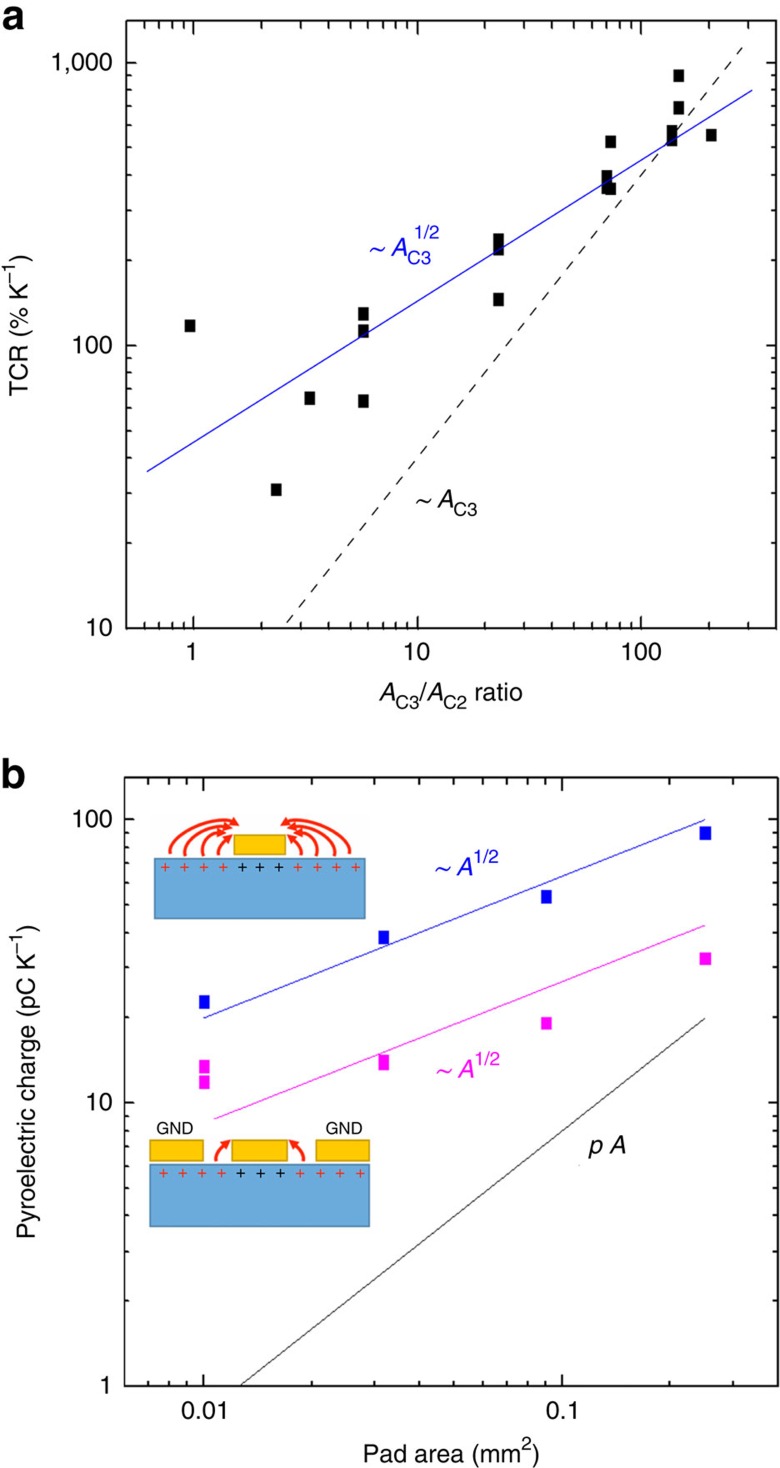
Scaling of device response with area. (**a**) TCR for 18 devices with different *A*_C3_/*A*_C2_ (*A*_C3_ varies with *A*_C2_ constant), extracted from thermo-electrical measurements as in Fig. 3b. For decreasing *A*_C3_, the TCR follows a square root law (blue line) instead of the linear dependence predicted by the model^2^ (black dashed line). (**b**) Integrated pyroelectric charge per *K* measured for unscreened (blue) and screened (magenta) Au pads on *z*-cut LN. A square root scaling law with area is found in both cases, which explains the behaviour observed in **a**. Even when screened, small pads still offer a significant enhancement of the pyroelectric charge compared with what expected from the model^2^ (∼*p A*, black line).

Finally, we discuss how our TCR scales with *A*_C3_/*A*_C2_. Figure 4a plots the measured TCR for 18 devices fabricated by keeping *A*_C2_ constant (22 × 20 μm^2^) and varying *A*_C3_ from 25 × 25 μm^2^ to 300 × 300 μm^2^. Under our design assumptions (and disregarding *C*_1_), the TCR should be proportional to the pyroelectric charge generated by *C*_3_. Hence, from the pyroelectric law of equation (1) one would expect a linear relation TCR ∼*A*_C3_. Our data, however, fit a square root dependence, indicated by the blue line. This behaviour cannot be explained by invoking the direct effect, whose contribution appears on a much smaller scale (TCR ∼5% K^−1^, see Supplementary Note 3). Rather, we have to consider that the pyroelectric substrate does not end at the pads edge. Such pads are thus not driven just by the crystal below them (as assumed by a linear dependence on area), but can also be affected by the exposed polarization of their surrounding areas (an effect scaling linearly with perimeter, hence the square root dependence on area). To better quantify this behaviour, we prepare Au pads of different sizes (0.01–0.3 mm^2^) on our pyroelectric substrates and measure the total charge generated on heating (Fig. 4b). This is accomplished by placing an electric probe on each pad, connecting the probe to ground and integrating the pyroelectric current flowing through the probe over the whole temperature ramp. In one case, the pads are kept isolated on the 1 × 1 cm^2^ pyroelectric surface and independent from each other. In another case, the whole surface around the pads is coated with Au and grounded during all measurements (with only a gap of 5 μm uncoated around the pads). This is meant to suppress any contribution from areas beyond the pad footprint. Figure 4b shows that a square root dependence on pad area is observed in both cases. The total pyroelectric charge decreases by a factor ∼2–3 on screening, but is still above what would be expected from the model^2^ *Q* Δ*T*^−1^=*p A* even for relatively large pads (using *P*=77 μC m^−2^ K, as measured for a LN sample fully covered with Au and consistently with literature^2^,^12^,^45^). This result has major technological implications, as it proves that a substantial contribution to the observed TCR enhancement for small *A*_C3_ arises within the first few micrometres from the pad edge. It is then possible to harvest an enhanced pyroelectric charge in a dense array of small pixels, with only a tiny gap of few micrometres separating two adjacent devices.

## Discussion

In principle, *A*_C3_/*A*_C2_ >10 is desirable, because it can deliver TCRs up to 900% K^−1^ (Fig. 4a and Supplementary Fig. 7), but this upscaling is bound by the maximum gate voltage variation allowed for the GFET (dynamic range). When probed electrically (Fig. 3a), our GFETs show no gate leakage up to ±5 V (∼5 MV cm^−1^). Beyond this value, dielectric breakdown can occur. This determines the maximum thermal shock a device can sustain without failing, inversely proportional to the TCR. However, this is not a concern if the environment *T* is drifting on a timescale much larger than the measurement timeframe, e.g., during a day/night indoor *T* cycle. Although internal pyroelectric leakage can be neglected over a few minutes, it can still discharge a device completely over longer timeframes. This will always leave the GFET at the best operating point to respond to sudden signals (see Supplementary Note 4). If one wants to scale down the pixel size while maintaining the same area ratio, the channel area must be decreased accordingly. As the 1/*f* noise scales with channel area^43^, we can expect the GFET noise to increase and cancel the benefit of a large TCR when the NEST is evaluated. However, Fig. 4 shows that the TCR scales sub-linearly with area. For pixels approaching the scale required for high-resolution IR cameras (20 × 20 μm^2^)^2^, it is better to make a large (several micrometres) channel and accept a lower area ratio (e.g., *A*_C3_/*A*_C2_ <10), because the small price paid in terms of TCR will be more than compensated by lower noise, a less critical lithographic process and a detector more resilient to sudden thermal shocks.

In conclusion, we presented a graphene-based pyroelectric bolometer operating at room temperature with TCR up to ∼900% K^−1^ for a device area ∼300 × 300 μm^2^ able to resolve temperature variations down to 15 μK at 1 Hz. For smaller devices, the TCR scales sub-linearly with area, due to an enhancement of the collected pyroelectric charge in close proximity to the metallic edges. When used as MIR PDs, our devices deliver very promising performance (in terms of responsivity, speed and NEP) even on bulk substrates and are capable to detect warm bodies in their proximity. Spectral selectivity can be achieved by patterning resonant structures as part of the pixel layout. This technology is competitive on a number of levels, ranging from high-resolution thermal imaging (small pixel limit) to highly sensitive spectroscopy in the MIR and far-IR (large pixel limit).

## Methods

### Device fabrication

**Figure 5:**
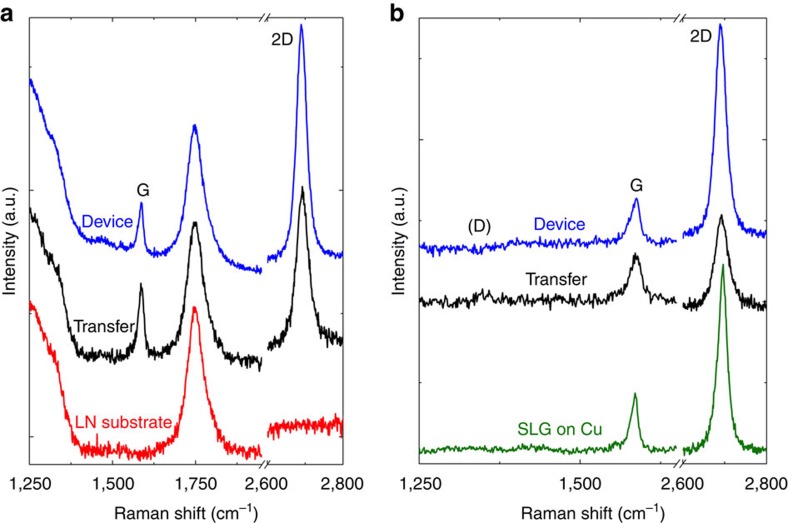
Raman spectroscopy. (**a**) Raman spectra of the bare LN substrate (red curve), of SLG transferred on LN (black curve) and of SLG on LN after device fabrication (blue curve). (**b**) Raman spectrum of the as-grown SLG on Cu (green curve), of SLG film transferred on LN (black curve) and of SLG on LN after device fabrication (blue curve) and subtraction of the substrate contribution.

SLG is grown by chemical vapour deposition on 35 μm-thick Cu following the process described in ref. 46. The quality of the material is monitored by Raman spectroscopy using a Renishaw InVia equipped with a × 100 objective (numerical aperture=0.85). We use an excitation wavelength of 514.5 nm and a laser power below 300 μW to avoid any possible damage. Figure 5b (green curve) shows the Raman spectra of SLG on Cu. The 2D peak is single-Lorentzian, a signature of SLG, with full width at half maximum (FWHM(2D))=26 cm^−1^ (ref. 47). The *D* to *G* intensity ratio, *I*(*D*)/(*G*), is ∼0.1, indicating a defect density ∼2.5 × 10^10^ cm^−2^ (refs 47, 48, 49, 50).

SLG is then transferred on the positively charged surface of *z*-cut LN (Roditi International Ltd) by spin coating a 500 nm layer of polymethyl methacrylate (PMMA) and then etching the Cu foil with an aqueous solution of ammonium persulfate^46^. The resulting SLG/PMMA film is rinsed in water and picked up with the target substrate. After drying, the sample is placed in acetone to dissolve the PMMA, leaving a film of SLG on LN. Figure 5a plots the Raman spectrum after transfer on LN (black curve). The spectrum for the bare LN substrate is also reported (red curve). The *D* peak region at ∼1,350 cm^−1^ is convoluted with a band at 1,200–1,450 cm^−1^ arising from optical phonons in LN^51^. An additional LN peak is also present at 1,744 cm^−1^, which does not overlap with any of the characteristic features of SLG^51^.

In Figure 5b we plot the transferred SLG spectrum (black curve) after subtracting the reference contribution of the LN substrate and normalization to the LN peak at 1,744 cm^−1^. The 2D peak is single-Lorentzian with FWHM(2D) ∼38 cm^−1^. The position of the *G* peak, Pos(*G*), is 1,588 cm^−1^, with FWHM(*G*) ∼14 cm^−1^. The position of the 2D peak, Pos(2D), is 2,691 cm^−1^. The 2D/G peak intensity and area ratios, *I*(2D)/*I*(*G*) and *A*(2D)/*A*(*G*), are 2.1 and 4.1, respectively, suggesting a doping concentration ∼5 × 10^12^ cm^−2^ (∼300 meV)^52^,^53^,^54^. The spectra show *I*(*D*)/(*G*)∼0.22, corresponding to a defect concentration ∼5.4 × 10^10^ cm^−2^ (refs 47, 48, 49, 50), similar to that before transfer, indicating that negligible extra defects are introduced during the transfer process^47^,^48^,^49^,^50^.

The fabrication of top-gated GFETs on LN presents additional challenges compared with Si/SiO_2_. Owing to the pyroelectric nature of LN, a significant static charge can build on both surfaces. To preserve our devices from discharge-induced damage, we initially prepare all metallic features on the LN surface as an electrically connected pattern, i.e., source, drain and floating gate contacts of the GFET are shorted together by means of metallic lines. In this configuration, the device can undergo all the required high-*T* (up to 120 °C) processing steps without failing. The shorts are then removed in the last step, when no further heating is required aside from normal sensor operation.

**Figure 6:**
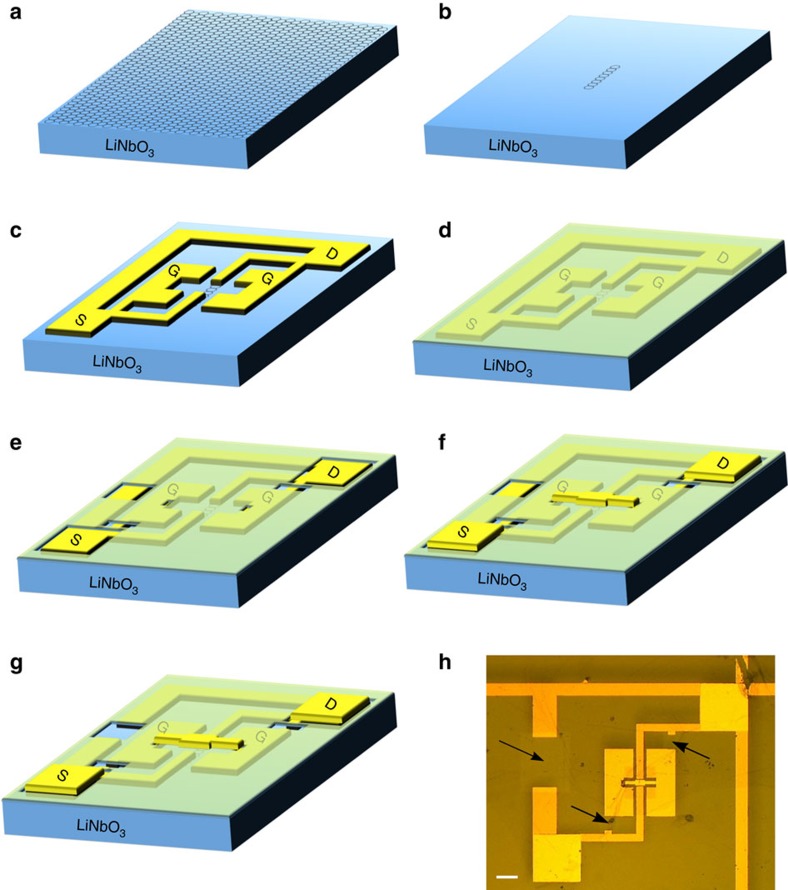
Device fabrication. (**a**–**g**) Step-by-step device fabrication process. (**h**) Optical image after device fabrication. Scale bar, 100 μm.

The complete device fabrication process is outlined in Fig. 6. First, SLG channels are patterned (Fig. 6b) using optical lithography and dry etching in O_2_ (20 W for 20 s). A second lithographic step defines the metal contacts (source and drain), as well as the floating gate pads directly in contact with the substrate (Fig. 6c). All features are shorted together as explained above. Before the deposition of a 40 nm-thick Au layer via thermal evaporation, a mild Ar plasma (Moorfield NanoETCH, 0.5 W, 20 s) is used on the exposed SLG areas. This is crucial to achieve a good contact resistance (<100 Ω), as defects induced by the plasma ensure a good bonding with the metal^55^. Further, a 10 nm Al_2_O_3_ layer is deposited by atomic layer deposition at 120 °C, to serve as gate dielectric (Fig. 6d). Two nm of Al are used as a seed layer for atomic layer deposition^56^. Optical lithography is again used to define apertures in the Al_2_O_3_, to expose the contact pads (source and drain), part of the shorting lines and a small section of the lateral pads where the top electrode needs to be anchored. The Al_2_O_3_ is then wet-etched in an alkaline solution (D90:H_2_O 1:3) for ∼6 min, leaving the structure in Fig. 6e. Another lithographic step is then used to finalize the top-gate via thermal evaporation and lift-off of 2/60 nm of Cr/Au. Bonding pads are also prepared in this step, overlapping those deposited with the contacts (Fig. 6f). Finally, the electrical shorts are removed with a lithographic step followed by wet-etching of the Au lines in an aqueous solution of KI:I_2_ (Fig. 6g). An optical picture of the final device is shown in Fig. 6h, the arrows indicating where the Au shorts have been etched.

### Thermo-electrical characterization

*T*-dependent electrical characterization is performed with a Cascade probe station with a *T*-controlled chuck, coupled to an HP4142B source meter. The spectral density of the current fluctuations (*S*_I_) is the Fourier transform of the drain current recorded during 100 s with a sampling of 1 ms. The normalized *S*_I_ *I*^−2^ exhibits the same *f*^−1^ dependence for all drain voltages applied.

### Device characterization

Devices are illuminated by a linearly polarized quantum cascade laser with a frequency range from 1,000 to 1,610 cm^−1^ (∼6.2–10 μm) scanned using a motorized *xyz* stage. The laser is modulated using a chopper and the current measured using a current pre-amplifier and lock-in amplifier. The light polarization is controlled with a ZnSe wire grid polarizer. The light is focused using ZnSe lenses with numerical aperture ∼0.5. The power for each frequency is measured using a bolometric power meter and the photocurrent spectra are normalized by this power to calculate the responsivity.

### Simulations

Optical calculations are performed with a finite-difference time-domain method^57^,^58^ assuming an infinite array of infinitely long Au fingers (40 nm-thick) on top of a semi-infinite LN substrate. Thermal transient calculations are performed with a finite element method (see www.comsol.com) assuming a 500 μm-thick LN substrate on top of a 3 mm-thick Au block (heat-sink) whose back surface is kept fixed at RT. More details can be found in Supplementary Notes 1 and 2.

### Data availability

All data generated or analysed during this study are included in this published article (and its Supplementary Information files).

## Additional information

**How to cite this article**: Sassi, U. *et al*. Graphene-based mid-infrared room-temperature pyroelectric bolometers with ultrahigh temperature coefficient of resistance. *Nat. Commun.* **8**, 14311 doi: 10.1038/ncomms14311 (2017).

**Publisher's note**: Springer Nature remains neutral with regard to jurisdictional claims in published maps and institutional affiliations.

## Supplementary Material

Supplementary Information Supplementary Figures 1-8, Supplementary Notes 1-6 and Supplementary References
