# Supplementary material for: Graphene-based mid-infrared room-temperature pyroelectric bolometers with ultrahigh temperature coefficient of resistance
Source: Nat Commun. 2017 Jan 31;8:14311. doi: 10.1038/ncomms14311 (PMC5290316; doi:10.1038/ncomms14311)
Supplement: Supplementary Information — Supplementary Figures 1-8, Supplementary Notes 1-6 and Supplementary References [file ncomms14311-s1.pdf]

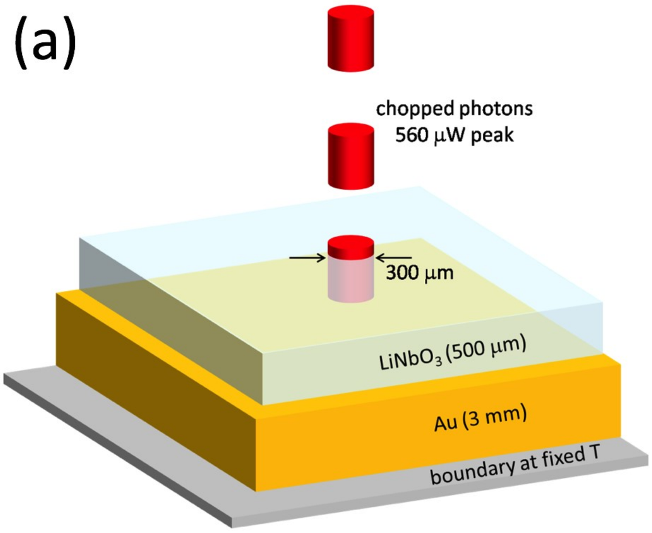

	$LiNbO_3$	*Au*	
mass density	4650	19300	kg/m³
capacitance	633	129	J/kg-K
conductivity	5.6	318	W/m-K

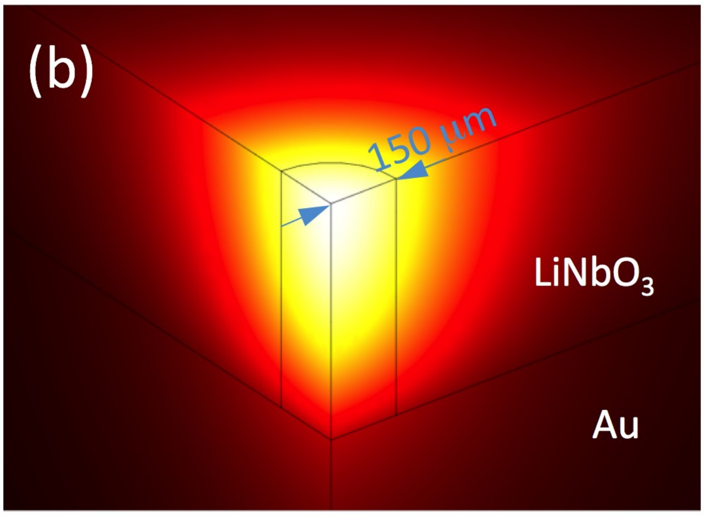

Supplementary Figure 1. (a) Model used for simulations and associated parameters. **(b)** Simulated temperature distribution around the 150-μm-radius laser spot (white=hotter, red=colder).

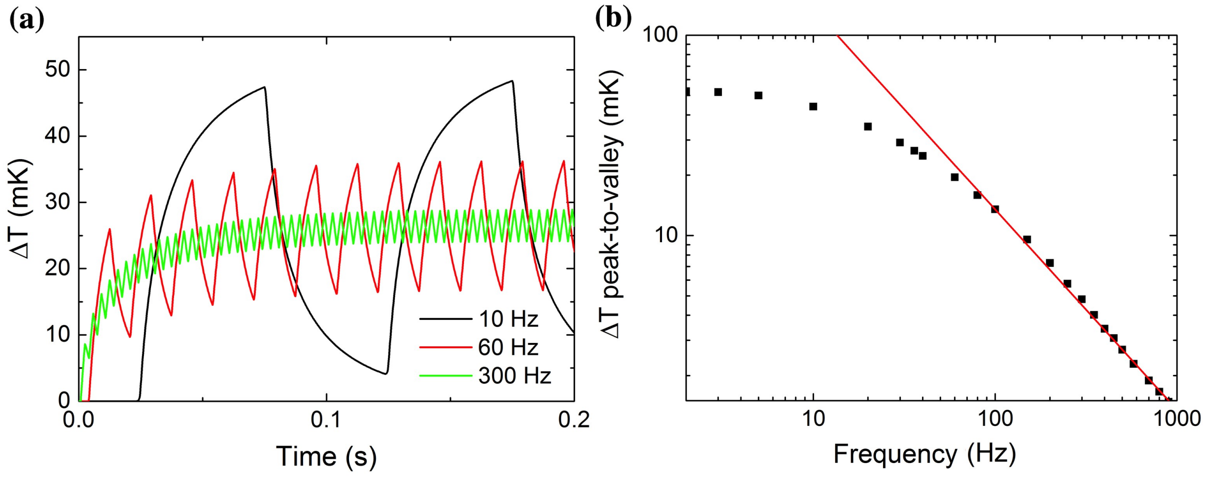

Supplementary Figure 2. (a) Total T transient for 3 chopper frequencies. **(b)** Δ*T* peak-to-valley dependence as a function of the chopper speed.

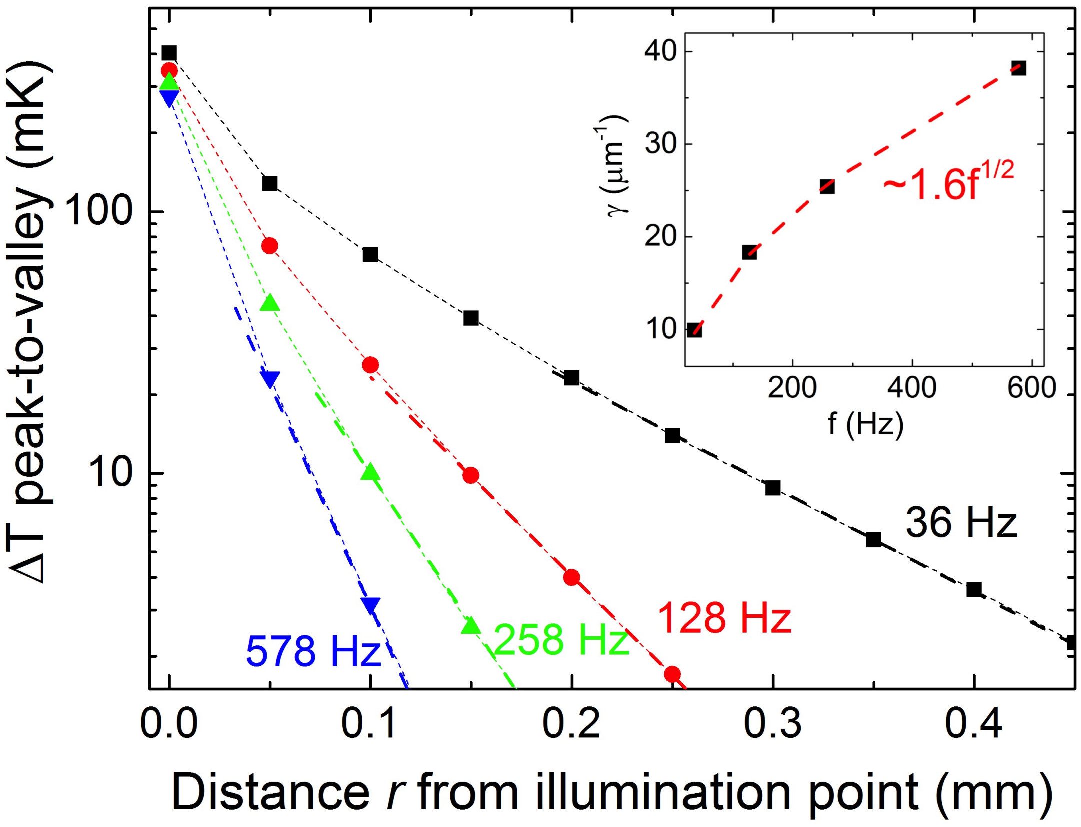

Supplementary Figure 3. Radial decay of Δ*T* peak-to-valley for different chopper speeds. A decay of the form ~exp(−γ*r*) is found. Inset: Dependence of γ from the chopper frequency *f*.

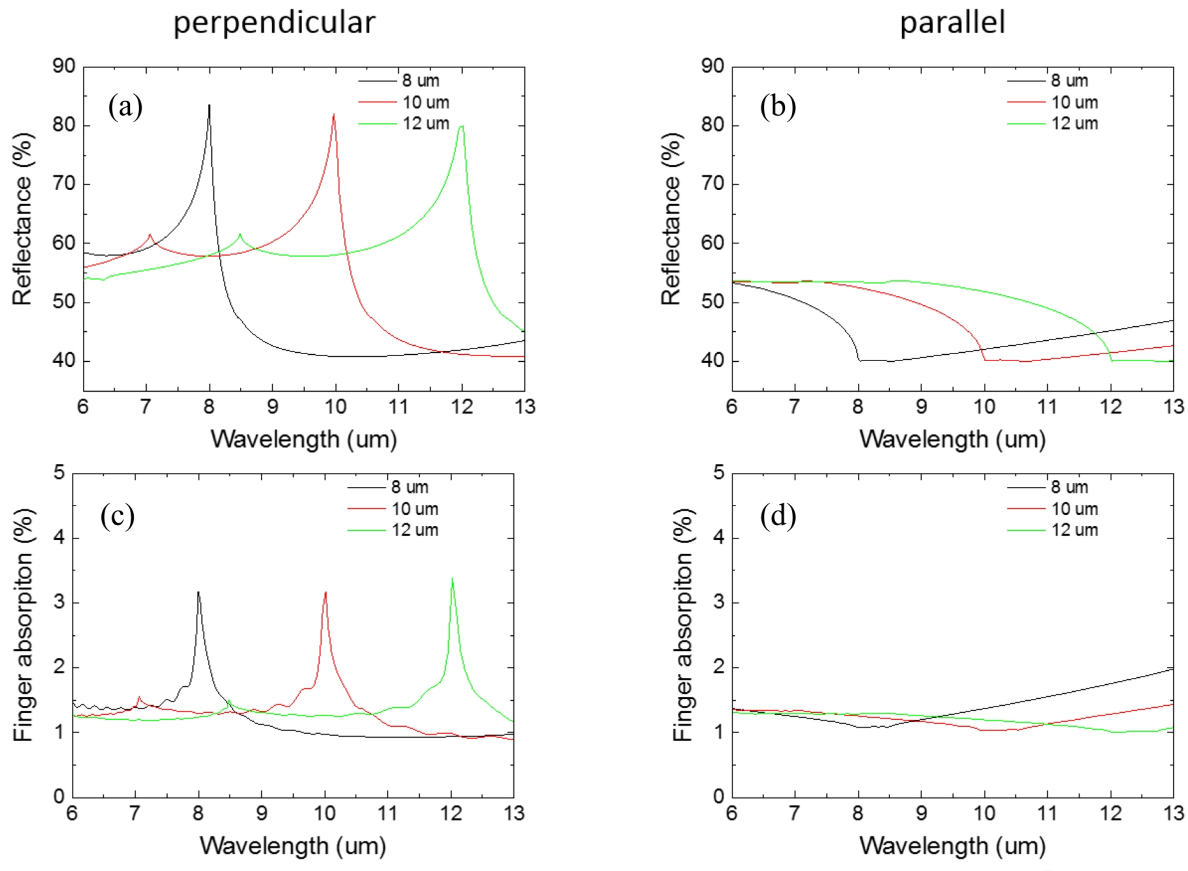

Supplementary Figure 4. Simulated reflectance and absorption in the Au fingers for perpendicular (a,c) and parallel (b,d) polarized light and different finger pitch.

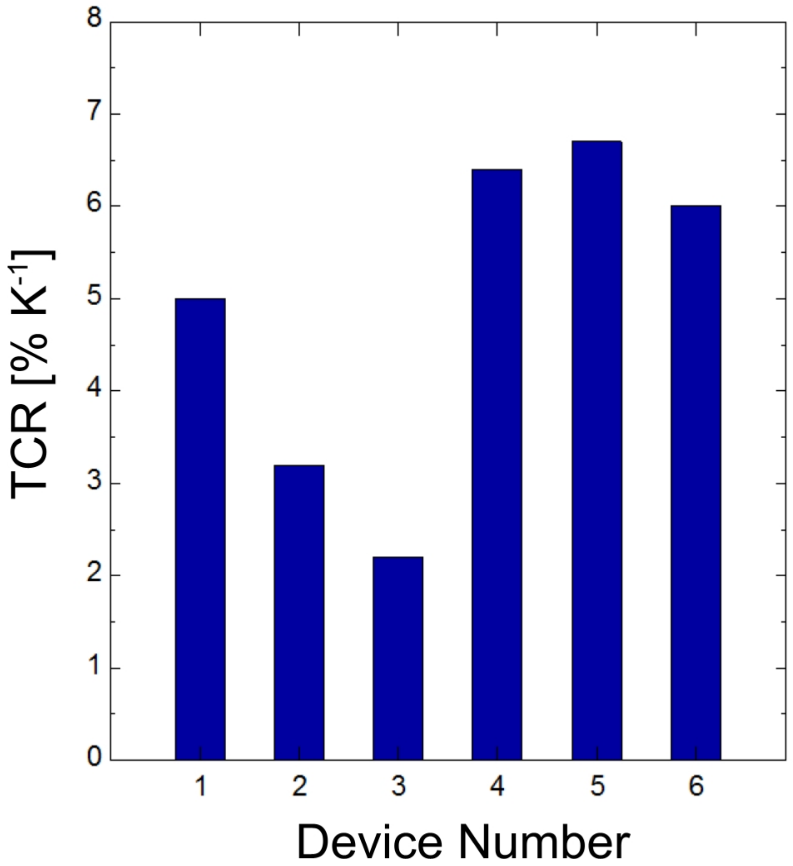

Supplementary Figure 5. Measured TCR for graphene devices on LN exploiting the direct effect only.

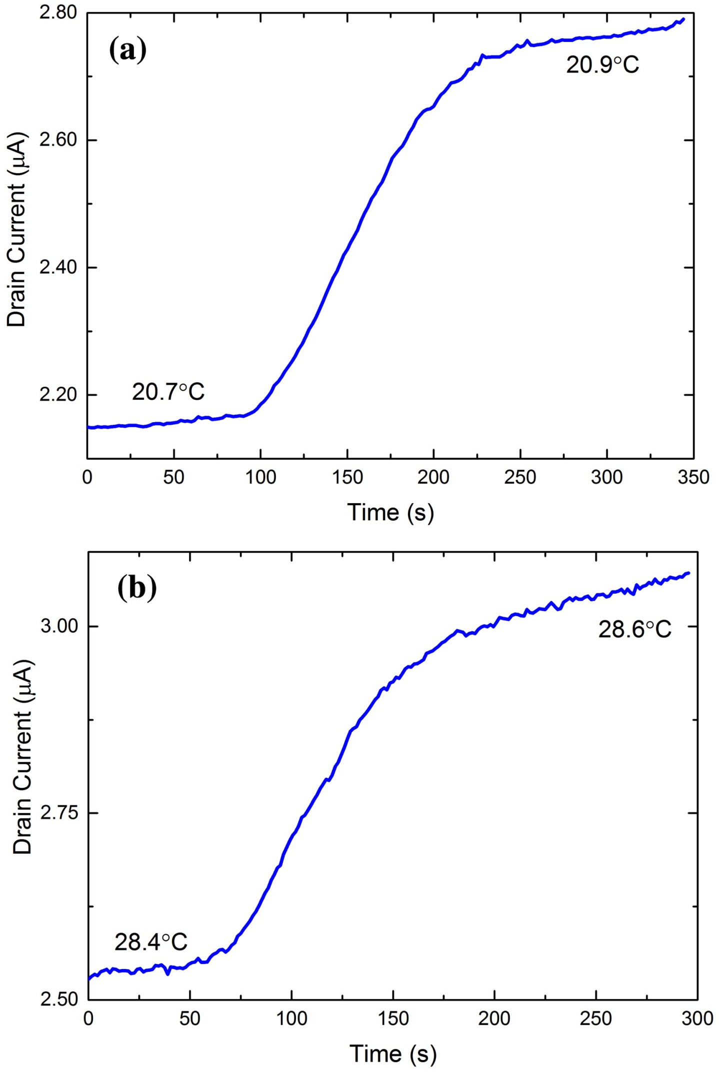

Supplementary Figure 6. Drain current response for the same device upon (**a**) T increase of 0.2 °C from RT and (**b**) after a slow 8 °C heating ramp at 2 °C per hour.

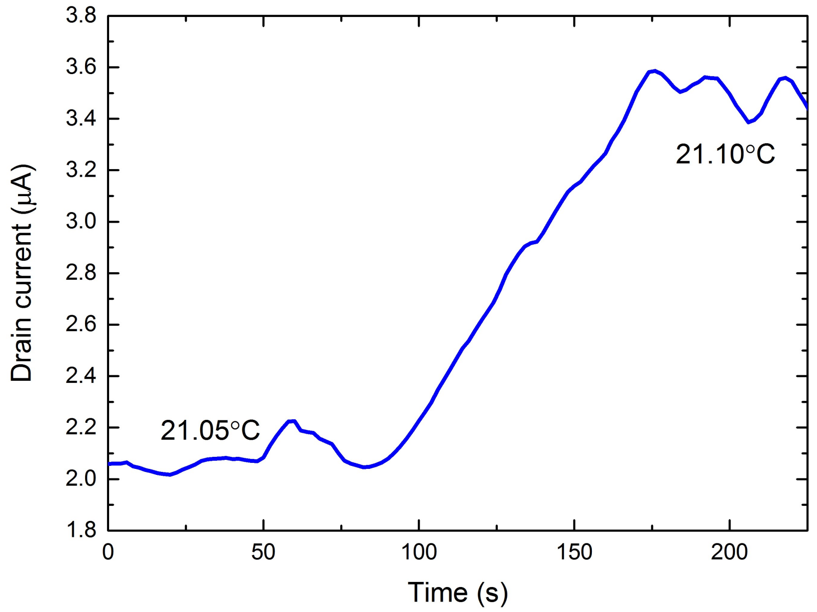

Supplementary Figure 7. Drain current response for a 300x300 μm^2 pixel upon a 0.05 °C T increase.

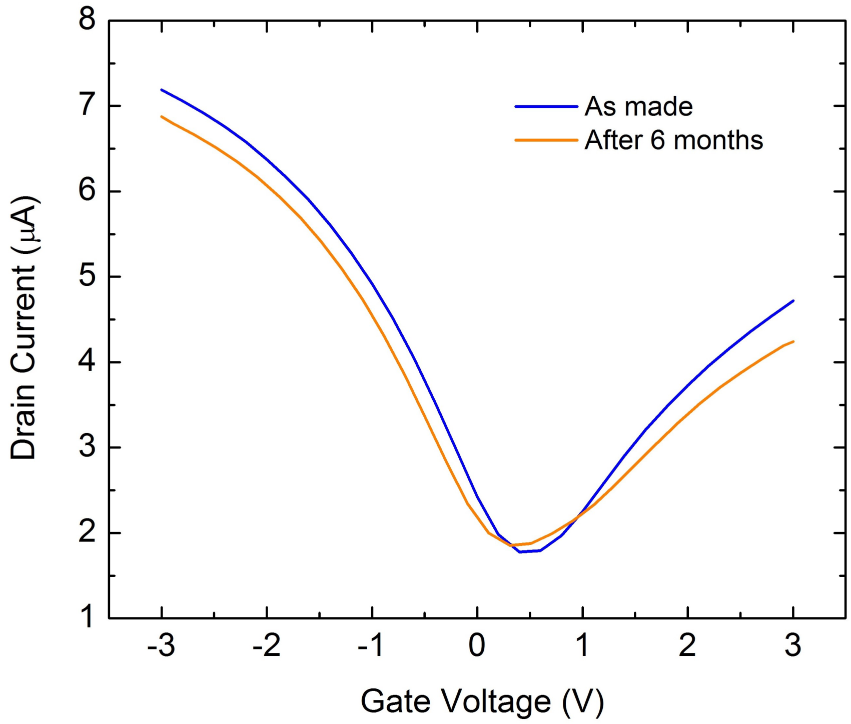

Supplementary Figure 8. Stability of a typical device after 6 months in ambient conditions.

Supplementary Note 1: Thermal model

Fig. 2e in the main text shows that, when a chopper is used to induce a periodicity in the illumination (and associated heating), the amplitude of the resulting periodic photoresponse scales linearly with the inverse of the chopper frequency. We present here a thermal model and associated simulations to explicitly study the time-dependent heating and cooling properties of our device and confirm the above conclusion.

Supplementary Fig. 1a illustrates how we model the experiment in Fig. 2e. A laser beam with equivalent characteristics (560 μW, 300 μm in diameter) is chopped at variable frequencies with a 50 % duty cycle. The 500-μm-thick LN substrate is in contact with a 3-mm-thick Au layer (that represents the chip carrier), acting as heat sink towards a boundary at fixed temperature T_0. Typical materials parameters are reported in Supplementary Fig. 1a [1]. We make the following approximations:

- We assume radiation is fully and uniformly absorbed in the LN film;
- We disregard heating losses by convection and radiation.

Under these assumptions, the qualitative temperature distribution near the laser spot for an arbitrary instant in time is shown in Supplementary Fig. 1b.

Supplementary Fig. 2a shows the simulated temperature transient $\Delta T = T - T_0$ for the point at the top of the LN film and at the centre of the illumination spot. The average T saturates at $\sim T_0 + 25$ mK for all chopper frequencies, because this would be the equilibrium value for constant illumination at 280 μW (the impinging power is 560 μW / 2 at all frequencies for a symmetric chopper). The photoresponse measured by the lock-in in Fig. 2e overlooks this average ΔT and it only reflects the peak-to-valley ΔT of a single period, in phase with the chopper. This, on the other hand, varies significantly in Supplementary Fig. 2a for the three frequencies reported.

The full dependence of the ΔT peak-to-valley from the chopper frequency f is shown in Supplementary Fig. 2b. The calculations are performed with a finite element method (FEM)[2] using the assumptions made for Supplementary Fig. 1. Despite the approximations used, our simulations reproduce the f^{-1} behaviour observed experimentally above 60 Hz in Fig. 2e. For low frequencies (< 10 Hz), the system saturates within one period.

To match the conditions used for the photomapping in Fig. 2a-d, we now reduce the spot diameter to 10 μm and increase the laser power to 1.8 mW. Supplementary Fig. 3 shows the ΔT peak-to-valley for a number of points on the LN surface as a function of their distance r from the centre of the illumination spot. Outside the illuminated spot, ΔT decays with a rate

that depends on the chopper frequency. A decay of the form ~exp(−γr) is found, with $\gamma = 1.6$ $f^{1/2}$. In practice, this means that the higher the chopper frequency, the less the heat delivered by the laser in one cycle is allowed to spread radially. For this reason, the photomapping in Fig. 2d is much more resolved than that in Fig. 2a.

Supplementary Note 2: Wavelength-selective absorption

Fig. 2f shows that the photoresponse of a device with lateral pads patterned as parallel fingers with pitch L and filling ratio 0.5 exhibits a peak in the ratio between parallel and perpendicular incident light. The spectral position of such peak is linked to the geometrical parameter L. Fig. 2g indicates that the total absorption (linked to the device photoresponse) shows peaks in the parallel/perpendicular ratio at wavelengths closely matching the experiment. However, Fig. 2g does not allow us to discriminate between reflectance, pattern absorption and substrate absorption, i.e. the individual components contributing to the total absorption.

In Supplementary Fig. 4 we plot the simulated reflectance and the simulated absorption in the Au fingers for both parallel and perpendicular polarized light. We show results for L = 8, 10, 12 μm, as in Figs 2f,g. For both reflectance and finger absorption, peaks are found at the corresponding wavelength. Reflectance and finger absorption are measured as % of the total incident light. This means that, even if more light is reflected at resonance for the perpendicular polarization, this is also the condition that results in maximum absorption in the fingers. However, peak absorption in the fingers at resonance is ~3 % for the perpendicular polarization (versus ~1 % off-resonance and for the parallel polarization), while the measured absorption of a 500-μm-thick LN substrate is ~75 % in this wavelength range. Hence, substrate absorption dominates for our devices, overshadowing the absorption features in Supplementary Fig. 4 and the total absorption is eventually determined by 1−Reflectance, as explained in the main text. In the limit of thin substrates, however, which is the case of highest technological interest, the photoresponse will strongly depend on the absorption in the fingers and simulations like that in Supplementary Fig. 4 will be crucial to optimize the design of patterns to enhance absorption and spectral selectivity.

Supplementary Note 3: Characterization of the direct effect

We now consider the conductivity modulation of graphene induced by the substrate (direct effect) in devices where the floating top-gate has been omitted. We deposit the Al_2O_3 dielectric layer in all cases, to improve device stability. We extract the TCR of such devices

by means of thermo-electrical measurements, as in Fig. 3b, but with T ramp expanded to 1 °C because of the weaker response.

Supplementary Fig. 5 plots the experimental TCR distribution across multiple devices with equivalent geometry. The average TCR (in module) is 4.9 % K^{-1}, on par with that reported by Supplementary Ref. 3 for SLG on lead titanate zirconate (~6 % K^{-1}). We conclude that the contribution of the direct effect to the device sensitivity is negligible compared to the amplified response achieved with the floating gate, which remains dominant even for the smallest pixel size (A_{C3}/A_{C2} = 1, see Fig. 4a). This means that, in most practical cases, there is little benefit in placing SLG in direct contact with the pyroelectric substrate. We envisage that the performance of our devices can be further optimized by implementing a suitable spacer between SLG and the pyroelectric substrate, chosen from materials known to provide a smooth and inert interface with SLG (e.g., boron nitride[4]).

Supplementary Note 4: Device resilience to large temperature variations

The breakdown field of the Al_2O_3 dielectric layer limits the dynamic range of the GFET for a given TCR. We consider safe to apply gate fields up to 5 MV cm^{-1}, corresponding to an applied voltage of ±5 V for a 10-nm-thick alumina film. A close inspection of Figs. 3a,b reveals that, if 0.2 °C induces a gate voltage of −0.44 V, a T variation > 3 °C may result in device failure, not acceptable for applications. However, all samples are subjected to much larger (> 10 °C) temperature variations over their lifespans and proved very resilient. We observed no failures and a consistent response irrespective of environment.

The reason for this behaviour resides in the T gradient, i.e. how slow or fast the device experiences the T variation. Supplementary Fig. 6a plots the response of a representative device with TCR ~ 130 % K^{-1} as T increases by 0.2 °C in 3 minutes. For this device, this corresponds to an induced gate voltage of −0.22 V (because of the different A_{C3}/A_{C2}, the TCR is roughly half of that in Fig. 3b). Accordingly, increasing T by 8 °C should result in an induced voltage of ~9 V, destroying the device. The T is thus raised by ~8 °C with a slow rate (2 °C per hour) and the device response to a T variation of 0.2 °C is measured again (Supplementary Fig. 6b). Interestingly, not only the device survives the dielectric breakdown, but its response is still consistent with Supplementary Fig. 6a. Indeed, the drain current at 28.4 °C (2.53 μA, Supplementary Fig 6b) is almost the same as previously measured at 20.8 °C (Supplementary Fig. 6a), indicating that the device operating point tends to drift in the opposite direction of the applied stimulus. This is not due to the

GFET hysteresis, as this would have been evident also in Fig. 3b (where no hysteresis is observed). Hence, we conclude that the drift preserving the device comes from the slow (hours) internal discharge of the pyroelectric crystal. This is negligible over a few minutes (Fig. 3b) but can play an important role over several hours.

Supplementary Note 5: Drain current response for large pixels and device stability

Here we report the maximum TCR measured on a 300x300 μm^2 pixel. By applying a drain voltage of 10 mV, the drain current is measured while changing T, as for Fig. 3b. However, due to the large TCR, we increase T by 0.05 °C (rather than 0.2 °C, as in Fig. 3b). Supplementary Fig. 7 reports the drain current response, showing a TCR of ~900 % K^{-1}.

We note that the electrical characteristics of our GFETs do not change with time. This is because the Al_2O_3 gate dielectric also works as passivation layer. In Supplementary Fig. 8 we show that the doping of an ideal GFET (slightly p-type immediately after device fabrication) does not vary after 6 months in ambient conditions, ensuring the long-term stability of the detector operating point.

Supplementary Note 6: Consistency of the electrical model

Qualitatively, the electrical model discussed in Fig. 1a,b is consistent with the behaviour seen experimentally in Fig. 3b. We present here a simple calculation to show that all data are also quantitatively consistent.

Fig. 4b shows that we can measure directly the charge (in module) generated by the $LiNbO_3$ substrate on pads of different sizes upon a 1 K T change. The pad (C_3) of the device in Fig. 3a is 0.01 mm^2 in area, and partially screened by the source and drain contacts, so it generates~9.8 pC K^{-1}. This falls between the trends in Fig. 4b for screened and unscreened pads. For a T increase of 0.2 K (the value used in Fig 3b), we expect this pad to generate 1.96 pC (9.8 pC K^{-1} * 0.2 K). The capacitance of the GFET top-gate (C_2) for the same device is~4 pF [$C_2 = \varepsilon_0\ \varepsilon_r\ A_{C2}\ t^{-1}$, where ε_0 and ε_r are the vacuum and relative permittivity ($\varepsilon_r \sim 9$ for Al_2O_3), t is the oxide thickness, and A_{C2} the area of the top-gate]. Hence, from Eq. 4 we should expect a gate voltage for the SLG channel V_{TG} = 1.96 pC / 4 pF ~ 0.5 V. This is very close to the value measured in Fig. 3 (0.44 V), and is thus evidence that the model discussed in Fig.1b is correct and that there are no significant parasitic capacitances disrupting the predicted device operation.

Supplementary References

[1] See datasheets at http://www.roditi.com/ and www.lambdaphoto.co.uk.

[2] https://www.comsol.com

[3] Hsieh, C. et al. Graphene-lead zirconate titanate optothermal field effect transistors. *Appl. Phys. Lett.* **100**, 113507 (2012).

[4] Dean, C. R. et al. Boron nitride substrates for high-quality graphene electronics. *Nat. Nanotech.* **5**, 722-726 (2010).
